# Temperature- and Pressure-Dependent Calibration Transfer Bias of WMS, FWMS, and DAS Methane Retrievals near 1654 nm

**DOI:** 10.3390/s26196158

**Published:** 2026-09-28

**Authors:** Murat Guder, Ibrahim T. Ozdur

**Affiliations:** Department of Electrical and Electronics Engineering, TOBB University of Economics and Technology, 43 Sogutozu Caddesi, Ankara 06560, Turkey; mguder@etu.edu.tr

**Keywords:** tunable diode laser absorption spectroscopy, methane sensing, wavelength modulation spectroscopy, fixed-wavelength modulation spectroscopy, direct absorption spectroscopy

## Abstract

**Highlights:**

**What are the main findings?**

- WMS, FWMS, and DAS temperature–pressure sensitivities were compared for CH_4_ sensing.
- FWMS achieved the lowest RMSE (10.5%) when pooled over all 35 simulated operating points, whereas DAS performed best in the closed-volume scenario.

**What are the implications of the main findings?**

- A calibration transferred away from its reference state requires explicit temperature and pressure compensation.
- Method selection should reflect the required update rate, line center tracking, and baseline stability.

**Abstract:**

Temperature and pressure changes can introduce substantial bias when a tunable diode laser absorption spectroscopy calibration is used away from its reference condition. Here, scanned wavelength modulation spectroscopy (WMS), fixed-wavelength MS (FWMS), and integrated area direct absorption spectroscopy (DAS) are compared for the CH_4_ R(3) manifold near 1654 nm. To isolate the behavior of the retrieval methods, all three simulations use the same HITRAN2020 line-by-line model, optical depth, laser model, target geometry, and calibration state. The analysis covers temperature variation at constant pressure, pressure variation at constant temperature, and coupled temperature–pressure variation in a closed volume. With temperature held constant, the FWMS bias varies from −5.2% to −0.8% over 0.7–1.1 atm. At constant pressure, it changes from +23.4% to −9.8% between −20 and +40 °C. DAS gives the smallest endpoint bias in the closed-volume case, ranging from +19.1% to −8.0%. Across all 35 operating points, the RMSE is 10.5% for FWMS, 13.4% for WMS, and 14.9% for DAS. The pooled ranking is unchanged for modulation indices between 1.6 and 3.0 and for DAS integration windows between ±10 and ±100 GHz.

## 1. Introduction

Tunable diode laser absorption spectroscopy (TDLAS) is widely used for path-integrated methane measurements based on the Beer–Lambert law [1,2,3]. Established retrieval approaches include scanned wavelength modulation spectroscopy (WMS) [4,5,6], fixed-wavelength MS (FWMS) [1,7,8], and direct absorption spectroscopy (DAS) [9,10]. A calibration established at one temperature and pressure does not transfer unchanged to another thermodynamic state because both the line intensity and line shape vary. For methane near 1654 nm, earlier studies reported uncorrected temperature-dependent errors of about 20% for WMS and 30% for DAS over 263–323 K; pressure-dependent errors reached 3.69% over 0.6–1.1 atm [11]. Errors exceeding 50% have also been observed before temperature compensation [12]. Previous studies have developed method-specific corrections [4,5,7,9,12,13,14,15] and compared multiple retrieval approaches [11], while a recent review surveys compensation across sensor technologies [16]. The present contribution is a controlled comparison of three specified metrics: scanned WMS peak amplitude, fixed carrier FWMS amplitude, and integrated area DAS. Using the same HITRAN line list, laser model, optical depth, geometry, and reference calibration, we quantify their uncompensated calibration transfer bias across common temperature–pressure scenarios. This shared framework isolates the retrieval-dependent part of the calibration transfer bias from instrument-specific effects; the resulting comparison is numerical, conditional on the stated assumptions, and concerns the retrieval metrics rather than complete experimental instruments.

This study compares the three retrievals for the CH_4_ R(3) manifold near 1654 nm. The HITRAN line list, optical depth, laser model, target geometry, and reference point (293 K, 1 atm, and 1000 ppm·m) are kept identical so that differences can be attributed to the retrieval itself. We consider temperature variation at P = 1 atm, pressure variation at T = 293 K, and coupled temperature–pressure variation in a closed volume. Methane sensors are deployed under substantial ambient variability, from ambient and open-path monitoring [17] to oil and gas facilities [18,19] and coal mines [20,21,22]. Reported conditions include field sensor design envelopes of −30 to +70 °C and roughly 0.89–1.04 atm at oil and gas sites [18], deep mine airway pressures up to 1.10 atm [20], and above-atmospheric pressures with temperatures beyond +40 °C in below-sea-level basins [23]. The selected ranges, −20 to +40 °C and 0.7–1.1 atm, cover a representative subset of these reported conditions, and the sub-atmospheric limit additionally accommodates operation at elevated or high-altitude sites [14]. Throughout the paper, error refers to the uncompensated bias that results when the reference calibration is applied at a different thermodynamic state. This bias is one term in the uncertainty budget of a practical instrument; detector noise, etalon fringes, laser drift, and baseline reconstruction add further terms that are not simulated here but are discussed, together with the compensation strategies used in field sensors, in Section 7.2. FWMS performs best in the constant-pressure and constant-temperature cases, whereas integrated area DAS gives the lowest error for the closed volume. Taken over all operating points, the RMSE values are 10.5% for FWMS, 13.4% for WMS, and 14.9% for DAS.

## 2. Absorption Spectroscopy Theory

### 2.1. Beer–Lambert Law and Double-Pass Geometry

The open-path double-pass arrangement used in the model is sketched in Figure 1. A compact transceiver launches the beam through a methane plume of single-pass length L. After diffuse reflection from a Lambertian-like target, the return beam crosses the plume a second time and is collected by a co-located photodetector, giving an absorption path of 2 L. Target reflectivity, collection efficiency, and wavelength-independent optical loss are grouped into an effective return coefficient ρ_eff_ [5,24]. Because this coefficient multiplies the detector signal at every modulation phase, it cancels upon 1f normalization in both WMS and FWMS. The detected intensity can therefore be written as

*I_det_*(*t*) = *ρ_eff_* · *I*_0_(*t*) · *exp*[−2*α_ν_*(*t*) *L*] (1)

In Equation (1), α_ν_ denotes the absorption coefficient at optical frequency ν, and L is the one-way path length; the factor of 2 accounts for the return pass. The model treats ρ_eff_ as deterministic and wavelength-independent, so it also cancels from the 2f/1f ratio. This cancellation may not hold exactly in an experiment, where speckle, etalons, target inhomogeneity, or an angle-dependent return can perturb the signal. Those effects are outside the scope of the present analysis.

For the reference state (T = 293 K, P = 1 atm, X_CH4_ = 1000 ppm, and L = 1 m), the calculated peak double-pass absorbance is 0.078. This is close to the optically thin regime (A < 0.1), but the peak absorbance reaches 0.097 at −20 °C in the constant-pressure case. The simulations therefore retain the full exponential Beer–Lambert relation rather than its linear approximation [1,5,25].

### 2.2. HITRAN Line-by-Line Absorption Calculation

The HITRAN2020 calculation uses a fixed list of 30 transitions selected within ±25 GHz of 6046.944 cm^−1^ (1654 nm, 181,282.82 GHz), using their pressure-shifted centers at the reference state [26]. The same list is retained throughout the temperature–pressure sweeps. This spectral region contains the strong CH_4_ 2ν_3_ R(3) manifold and is accessible with InGaAs detectors and telecom-grade DFB lasers [17,18]. The nearest strong H_2_O features lie more than 1.6 cm^−1^, or roughly 48 GHz, from the methane line center and are considerably weaker at the reference state [26]. Water vapor interference is consequently neglected in the simulations, although it may become relevant for humid conditions or long optical paths.

(2) $$\alpha_{\nu }=N_{{\mathrm{CH}}_{4}}\;\cdot \;\sum_{i}S_{i}(T)\;\cdot \;\varphi_{V}\left(\nu -\nu_{0,i}\right)$$

Here, N_CH4_ is the methane number density, S_i_(T) is the temperature-dependent line intensity, and φ_V_ is the area-normalized Voigt profile for transition i, centered at the pressure-shifted line position ν_0,i_.

The total absorbance follows from the sum of the Voigt contributions of all HITRAN transitions inside the scan window. For transition i, the double-pass absorbance is

*A*_2*L*,*i*_(*ν*) = 2 *L S_i_*(*T*) *N_CH_*__4__ *φ_V_*(*ν* − *ν*_0,*i*_) (3)

where φ_V_ is the area-normalized Voigt profile introduced in Equation (6). Summing the individual terms gives the total double-pass absorbance

(4) $$A_{2L}(\nu )=\sum_{i=1}^{N_{\mathrm{lines}}}A_{2L,i}(\nu )$$

In Equation (4), N_lines_ is the number of transitions in the ±25 GHz channel plotted in Figure 2. The channel contains 30 transitions at the reference state, yet nearly all of the signal comes from the three dominant R(3) lines. Together, they contribute 99.9% of the peak absorbance and 99.7% of the integrated area; the other 27 transitions appear only as a weak pedestal in the wings.

Line intensities S_0_ tabulated at the HITRAN reference temperature T_0_ = 296 K, which is distinct from the calibration reference T_ref_ = 293 K used elsewhere in this paper, are transferred to the gas temperature T according to [1,26]

(5) $$S(T)=S_{0}\;\cdot \;\frac{Q(T_{0})}{Q(T)}\;\cdot \;\exp\left[-c_{2}\;E\prime \prime \;\left(\frac{1}{T}-\frac{1}{T_{0}}\right)\right]\;\cdot \;\frac{1-\exp\left(-c_{2}\nu \;/\;T\right)}{1-\exp\left(-c_{2}\nu \;/\;T_{0}\right)}$$

Here, Q(T) is the total internal partition function of ^12^CH_4_ taken from TIPS2021 [27], c_2_ = 1.4388 cm·K is the second radiation constant, and E″ denotes the lower-state energy. The three multiplicative terms describe the partition function, Boltzmann population, and stimulated emission correction, respectively [1,26].

### 2.3. Voigt Line Shape Profile

A Voigt profile is assigned to each transition [1,5] and is evaluated numerically via the Faddeeva function w(z):

(6) V(x, σG, γL) = Re[w(z)]σG 2π

The argument is $z\;=\;(x\;+\;i\gamma_{L})/(\sigma_{G}\sqrt{2})$, with *x* = *ν* − *ν*_0,*i*_; γ_L_ and σ_G_ denote the Lorentz half-width and Gaussian standard deviation, respectively. Air broadening, self-broadening, the temperature exponent, and the pressure shift coefficient are read separately for each transition from HITRAN [26].

## 3. Temperature- and Pressure-Dependent Spectral Response

### 3.1. Constant-Pressure Scenario (P = 1 atm, T = −20 to +40 °C)

At 1 atm, increasing the temperature from −20 to +40 °C narrows the absorption feature from 4.39 to 3.82 GHz FWHM, as seen in Figure 3 and Figure 4a. The methane number density falls as 1/T (Figure 4b), and the accompanying reduction in collisional broadening outweighs the modest increase in Doppler width. Temperature also alters the line intensity: the peak absorbance decreases from 0.097 to 0.070 across the same range. Because pressure is fixed, the peak frequency changes very little (Figure 4c).

### 3.2. Constant-Temperature Scenario (T = 293 K, P = 0.7–1.1 atm)

The constant-temperature spectra are shown in Figure 5. As pressure rises, both the methane number density and Lorentzian width increase approximately linearly. Consequently, the FWHM grows from 2.91 GHz at 0.7 atm to 4.34 GHz at 1.1 atm (Figure 6a), while the peak absorbance remains confined to 0.076–0.078; the density increase is largely offset by pressure broadening. A smaller, but important, change occurs in the line position. Relative to the 1 atm calibration point, the peak shifts from +100 MHz at 0.7 atm to −33 MHz at 1.1 atm (Figure 6c). This shift detunes the fixed laser frequency slightly from the absorption line and therefore influences both WMS and FWMS.

### 3.3. Closed-Volume Scenario

For the closed-volume calculation, the amount of methane and the volume are fixed, so the number density remains constant and pressure follows *P*(*T*) = *P*_ref_ · *T*/*T*_ref_. The spectra in Figure 7 therefore reflect the temperature dependence of line intensity together with the coupled pressure–temperature dependence of Lorentz broadening. These two effects largely compensate: the FWHM changes only from 3.83 to 4.05 GHz over the full range (Figure 8a). The pressure-induced line shift is also modest, moving from about +45 MHz at −20 °C to −23 MHz at +40 °C (Figure 8c). This scenario is useful because it isolates changes in line intensity and line shape from changes in methane number density.

## 4. WMS Error Analysis

Scanned wavelength modulation spectroscopy combines a slow wavelength ramp with a fast sinusoidal modulation. The ramp carries the laser across the absorption feature, whereas the high-frequency modulation generates the harmonics detected by the lock-in amplifier. Concentration is inferred from the peak of the background-subtracted 2f/1f profile [4,5]; fitting the entire scanned 2f/1f line shape is an established alternative [9,28]. For a consistent comparison, WMS, FWMS, and DAS are all referenced to T = 293 K, P = 1 atm, and X_CH4_·L = 1000 ppm·m. Any reported error is thus a calibration transfer bias caused solely by a change in temperature or pressure.

### 4.1. WMS Theory: Ramp + FM Modulation

Figure 9 illustrates how the scanned WMS signal is formed for a single Voigt line. At each position of the slow ramp, the sinusoidal FM probes a narrow interval around the carrier frequency. Demodulation at 1f and 2f produces a ramp-resolved 2f/1f trace, whose maximum within the prescribed search window serves as the concentration metric.

A slow linear ramp and a fast sinusoidal component are applied to the DFB laser current. The resulting instantaneous optical frequency is

*ν*(*t*) = *ν_c_* + Δ*ν* · *s*(*t*) + *a cos*(*ω_m_ t*) (7)

Here, s(t) varies from −1 to +1, Δν is the one-sided ramp span, a = 4.373 GHz is the FM depth, and *ω_m_* = 2*πf_m_*. These parameters give a reference state modulation index *m* = *a*/HWHM of approximately 2.2.

*I*_0_(*t*) = *I*_0_ [1 + *i*_1_ *cos*(*ωmt* + *ψ*_1_) + *i*_2_ *cos*(2*ωmt* + *ψ*_2_)] (8)

The intensity modulation contains linear and nonlinear components with amplitudes i_1_ = 0.05 and i_2_ = 0.002. Their phase offsets relative to the FM are set to ψ_1_ = 30° and ψ_2_ = 60°. This two-component IM description follows the standard WMS formulation [1,5], in which the FM–IM phase shifts are device-specific quantities to be characterized for each laser; reported magnitudes range from a few tens of degrees at kilohertz modulation frequencies [29] to values of order π for some DFB lasers [4]. Rather than fitting a particular device, these values represent a typical current-modulated DFB laser. The numerical model uses f_m_ = 50 kHz, an FM depth of 4.373 GHz, a ±15 GHz slow scan, a ±3 GHz WMS peak search interval, and a ±25 GHz spectroscopy window sampled every 10 MHz. One complete modulation period is evaluated at each ramp position, with 2048 samples per period. Collisional widths are calculated transition by transition, and DAS is evaluated on the same frequency grid.

Harmonic signals are obtained by digital lock-in demodulation [1,5,30,31]. The detector output is multiplied by sine and cosine references at nf_m_ and then averaged over an integer number of modulation periods. This procedure yields the in-phase and quadrature terms, together with their phase-independent magnitude:

(9) $$X_{\mathrm{nf}}\;=\;\frac{2}{T_{\mathrm{period}}}\int_{0}^{T_{\mathrm{period}}}I_{\det}(t)\;\cos(\mathrm{n\omega mt})\;\mathrm{dt}$$

(10) $$Y_{\mathrm{nf}}=\frac{2}{T_{\mathrm{period}}}\int_{0}^{T_{\mathrm{period}}}I_{\det}(t)\;\sin(\mathrm{n\omega mt})\;\mathrm{dt}$$

(11) $$R_{\mathrm{nf}}=\sqrt{{X_{\mathrm{nf}}}^{2}+{Y_{\mathrm{nf}}}^{2}}$$

The quantities X_nf_ and Y_nf_ are the in-phase and quadrature components. At every ramp position, the detector signal is generated from Equation (1) using the frequency and intensity modulation defined in Equations (7) and (8). Repeating the demodulation along the ramp produces the WMS harmonic profiles.

For concentration retrieval, the measured and zero-methane 2f quadratures are normalized by their respective 1f magnitudes. The magnitude of their vector difference defines the retrieval metric:

(12) $$S\left(\nu_{0}\right)\;=\;\sqrt{{\left(\frac{X_{2f}}{R_{1f}}-\frac{{X_{2f}}^{0}}{{R_{1f}}^{0}}\right)}^{2}\;+\;{\left(\frac{Y_{2f}}{R_{1f}}-\frac{{Y_{2f}}^{0}}{{R_{1f}}^{0}}\right)}^{2}}$$

The superscript 0 denotes the zero-methane background; all components in Equation (12) are evaluated at the same carrier frequency ν_0_. Normalization precedes subtraction, following the vector background subtraction procedure of Rieker et al. [5]. The scanned WMS concentration proxy is the maximum of S(ν_0_) within the peak search interval *W* = [*ν_c_* − 3 GHz, *ν_c_* + 3 GHz]:

(13) $${\left(X_{{\mathrm{CH}}_{4}}\cdot L\right)}_{\mathrm{ret}}\;=\;\frac{\max_{\nu_{0}\in W}\;S_{\mathrm{meas}}\left(\nu_{0}\right)}{\max_{\nu_{0}\in W}\;S_{\mathrm{ref}}\left(\nu_{0}\right)}\;\cdot \;{\left(X_{{\mathrm{CH}}_{4}}\cdot L\right)}_{\mathrm{ref}}$$

The labels meas and ref identify the measurement and calibration states. Equation (13) normalizes the maximum of the measured background-subtracted metric to the maximum at the reference state; the peak frequencies need not coincide. The resulting path-integrated concentration is compared with the true X_CH4_·L, so the reported bias measures calibration transfer rather than absolute spectroscopic accuracy. FWMS uses the same S metric at the fixed carrier frequency, while DAS normalizes integrated absorbance.

A central design parameter is the modulation index, *m* = *a*/HWHM, because it sets the WMS-2f/1f response. A Lorentzian feature reaches its largest second harmonic amplitude near m = 2.2 [2,5], which is the value adopted here. The sensitivity of the results to this choice is examined in Section 7.1.

At the reference condition, collision broadening dominates the R(3) manifold: the Lorentzian FWHM is about 3.98 GHz, compared with a Doppler FWHM of 0.55 GHz. The profile is therefore nearly Lorentzian over the ±4.373 GHz modulation interval. Using the corresponding HWHM of approximately 1.99 GHz, m = 2.2 leads to a = 4.373 GHz; the 2f-optimal modulation depth grows with the collisional linewidth [32]. Changing m would alter the absolute harmonic amplitude, but it would not eliminate the line shape dependence on temperature and pressure. Because the linewidth itself varies between approximately 2.91 and 4.39 GHz across the operating envelope, the effective index a/HWHM already ranges from 2.0 (−20 °C, 1 atm) to 3.0 (0.7 atm, 293 K) within the sweeps.

### 4.2. Results

The scanned WMS retrievals are summarized in Figure 10, and each curve passes through 1000 ppm·m at the reference state. With pressure fixed at 1 atm, the retrieved value falls from 1324 ppm·m (+32.4%) at −20 °C to 882 ppm·m (−11.8%) at +40 °C. Holding temperature at 293 K produces a much smaller excursion, from 904 ppm·m (−9.6%) at 0.7 atm to 1008 ppm·m (+0.8%) at 1.1 atm. For the closed volume, the two endpoints are 1296 ppm·m (+29.6%) and 890 ppm·m (−11.0%).

## 5. FWMS Error Analysis

FWMS follows directly from the scanned WMS model after the slow ramp is removed and the carrier frequency is fixed at the reference line center. The intensity modulation model, lock-in calculation, and normalized vector background subtraction are unchanged. A fixed carrier metric is available every modulation period, allowing a higher update rate without a slow wavelength scan. In the single-depth, single-metric implementation evaluated here, changes in the line center are not independently tracked.

### 5.1. FWMS Theory: Single-Wavelength FM + IM Model

In the single-line example of Figure 11, the carrier remains at ν_c_ while the sinusoidal modulation sweeps from *ν_c_* − *a* to *ν_c_* + *a*. The normalized harmonic response depends on the line shape over this interval and is therefore sensitive to carrier detuning and line center drift. A fixed carrier does not imply that only one instantaneous optical frequency is sampled.

Setting Δν = 0 and s(t) = 0 in Equation (7) gives

*ν*(*t*) = *ν_c_* + *a cos*(*ω_m_t*) (14)

Under these conditions, the detector signal is periodic at f_m_ and may be expressed as

*I_det_*(*t*) = *ρ_eff_I*_0_[1 + *i*_1_*cos*(*ω_m_t* + *ψ*_1_) + *i*_2_*cos*(2*ω_m_t* + *ψ*_2_)] ×*exp*{−*A*_2*L*_[*ν_c_* + *acos*(*ω_m_t*)]} (15)

The first factor accounts for residual amplitude modulation, while the exponential is the exact Beer–Lambert transmission at the instantaneous frequency. For illustration, consider an isolated symmetric line with the carrier exactly at the line center, weak absorbance *A*_2*L*,peak_ ≪ 1, and linear intensity modulation (i_2_ = 0). In this additional idealized limit, the zero-absorption 2f background vanishes, and the leading-order 2f magnitude is

*R*_2*f*_(*ν_c_*) ≃ *ρ_eff_* · *I*_0_ · *A*_2*L*,*peak*_ · |*H*_2_(*m*)| (16)

where H_2_(m) is the second Fourier coefficient of the line shape sampled during one modulation cycle:

(17) $$H_{2}\left(m,\;x_{0}\;=\;0\right)\;=\;\frac{2}{T_{\mathrm{period}}}\int_{-T_{\mathrm{period}}/2}^{+T_{\mathrm{period}}/2}\varphi \left(x_{0}+m\;\cos\left(\mathrm{\omega mt}\right)\right)\;\cos\left(2\mathrm{\omega mt}\right)\;\mathrm{dt}$$

In Equation (17), φ(x) is the peak-normalized line shape evaluated at the normalized detuning *x* = (*ν* − *ν_c_*)/*Γ*, where Γ is the line HWHM, and x_0_ is the normalized carrier detuning. Exactly at the line center, x_0_ = 0. To obtain the normalized metric to first order in absorbance, the 1f denominator is retained at its zero-absorption value:

*R*_1*f*_(*ν_c_*) ≃ *ρ_eff_* · *I*_0_ · *i*_1_ (18)

The corresponding leading-order approximation to the metric in Equation (12) is

(19) $$S\left(\nu_{c}\right)\;≃\;\frac{A_{2L,\mathrm{peak}}\cdot \left|H_{2}\left(m\right)\right|}{i_{1}}$$

Equations (16)–(19) illustrate the roles of the peak absorbance and modulation index under the additional assumptions stated above. They are not used to compute the reported results. The simulations retain the full exponential transmission, both intensity modulation terms (including i_2_ = 0.002), their phase offsets, and numerical lock-in integration followed by Equation (12). For a Lorentzian line, |H_2_(m)| peaks near m = 2.2 [2].

FWMS is evaluated with a = 4.373 GHz and the same intensity modulation and lock-in settings as WMS. Its retrieval uses S at the fixed carrier ν_c_ instead of the maximum of the scanned S profile:

(20) $${\left(X_{{\mathrm{CH}}_{4}}\cdot L\right)}_{\mathrm{ret}}\;=\;\frac{S_{\mathrm{meas}}\left(\nu_{c}\right)}{S_{\mathrm{ref}}\left(\nu_{c}\right)}\;\cdot \;{\left(X_{{\mathrm{CH}}_{4}}\cdot L\right)}_{\mathrm{ref}}$$

The fixed carrier metric is normalized to its reference state value. This single-depth, single-metric retrieval does not independently estimate pressure-induced line center motion. Its response depends on the line shape sampled across the modulation interval and on detuning of the carrier from the absorption peak.

### 5.2. Results

Figure 12 summarizes the FWMS results. At 1 atm, the retrieved concentration decreases from 1234 ppm·m (+23.4%) at −20 °C to 902 ppm·m (−9.8%) at +40 °C. When temperature is held at 293 K, the response is considerably flatter: 948 ppm·m (−5.2%) at 0.7 atm and 992 ppm·m (−0.8%) at 1.1 atm. The closed-volume endpoints are 1245 ppm·m (+24.5%) and 902 ppm·m (−9.8%).

## 6. DAS Error Analysis

Unlike the two modulation-based methods, direct absorption spectroscopy does not require harmonic demodulation. The laser is scanned through the absorption feature, transmission is converted to absorbance, and the area under the line provides the concentration metric [9,33]. If the entire feature is recorded against a perfectly known baseline, this area is independent of Voigt width and pressure shift. Real measurements have a finite scan span and an imperfect baseline, however, and both can introduce additional bias. Section 6.3 and Section 6.4 examine these two effects.

### 6.1. DAS Theory: Direct Absorption and Integrated Area Retrieval

The sequence used for DAS is illustrated in Figure 13. After the laser scans the line, the measured transmission is divided by a reconstructed baseline and converted to absorbance. Provided that the complete line is captured, the integrated area scales with S(T)N_CH4_L. Concentration then follows from the ratio between the measured and reference areas.

Two features distinguish DAS from FWMS: the laser scans the full absorption line rather than remaining at the line center, and the retrieval uses integrated absorbance instead of harmonic amplitudes [1,2,3,7,9]. For the double-pass arrangement in Figure 1, the detector signal is

*I_det_*(*ν*) = *ρ_eff_* · *I*_0_(*ν*) · *exp*[−2*α_ν_ L*] (21)

The terms ρ_eff_, I_0_(ν), and α_ν_ have the definitions given in Section 2. In an experiment, the product ρ_eff_·I_0_(ν) may be reconstructed from absorption-free portions of the scan [10,33]. Dividing by this baseline gives the double-pass absorbance

(22) $$A(\nu )=-\ln\left[\frac{I_{\det}(\nu )}{\rho_{\mathrm{eff}}\;\cdot \;I_{0}(\nu )}\right]=2\alpha_{\nu }\;L$$

For an integration half-width Ω centered at ν_c_, a line area capture factor ηᵢ(Ω,T,P) accounts for the fraction of transition i inside the integration interval:

(23) Aint(T,P)=∫νc−Ωνc+ΩA(ν) dν=2L NCH4(T,P)∑iSi(T)ηi(Ω,T,P)

The factor η_i_ is the fraction of transition i contained within the chosen window. It approaches unity as the window becomes infinite, at which point the integrated area no longer depends on Voigt width or pressure shift.

The retrieved path-integrated concentration is obtained by comparing the measured and reference areas:

(24) $${\left(X_{{\mathrm{CH}}_{4}}\cdot L\right)}_{\mathrm{ret}}=\frac{A_{\mathrm{int},\mathrm{meas}}}{A_{\mathrm{int},\mathrm{ref}}}\cdot {\left(X_{{\mathrm{CH}}_{4}}\cdot L\right)}_{\mathrm{ref}}$$

The reference state is T_ref_ = 293 K, P_ref_ = 1 atm, X_CH4_ = 1000 ppm, and L = 1 m. Equation (22) is integrated by the trapezoidal rule on the same frequency grid used to generate the HITRAN spectra.

### 6.2. Results

The DAS results in Figure 14 show a stronger pressure dependence than either modulation-based retrieval. At P = 1 atm, the inferred concentration ranges from 1368 ppm·m (+36.8%) at −20 °C to 864 ppm·m (−13.6%) at +40 °C. At 293 K, it increases from 711 ppm·m (−28.9%) at 0.7 atm to 1094 ppm·m (+9.4%) at 1.1 atm. The closed-volume calculation gives 1191 ppm·m (+19.1%) and 920 ppm·m (−8.0%) at the temperature endpoints. The strong pressure dependence in the constant-temperature sweep has a simple physical origin. Because the mole fraction is fixed at 1000 ppm, the methane number density scales with pressure, and the retrieved 711 ppm·m at 0.7 atm is close to the ideal gas expectation of 700 ppm·m; dividing the retrieved value by *P*/*P*_ref_ gives 711/0.7 ≈ 1016 ppm·m, leaving a pressure-corrected bias of about +1.6% due to the larger fraction of the narrower line inside the ±25 GHz window. Most of the DAS bias in this scenario is therefore the *N_CH_*__4__ ∝ *P* term rather than a line shape effect, and this density contribution can be corrected using a pressure reading when the temperature is known. The much weaker pressure dependence of WMS and FWMS reflects a partial cancellation between the density increase and pressure broadening at m ≈ 2.2.

### 6.3. Finite Window Convergence of the DAS Retrieval

A ±25 GHz interval is used for the DAS integration. Expanding the interval from ±10 to ±100 GHz increases the absolute integrated area by 5–14% as more of the Lorentzian wings are collected. The calibration transfer bias, however, changes by less than 1 percentage point between ±25 and ±100 GHz. Most of the truncation is common to the measured and reference spectra and disappears when their ratio is taken. Table 1 lists the DAS RMSE for half-widths between ±5 and ±100 GHz, together with the fraction of the ±100 GHz reference area captured by each window. Between ±10 and ±100 GHz, the pooled RMSE moves only from 14.4% to 15.1%, and the ordering of the three scenarios is preserved. A very narrow window behaves differently: at ±5 GHz, the retrieval approaches a peak height measurement, in which pressure broadening partly offsets the change in number density, and the pooled RMSE decreases to 13.5%; if the recorded scan were also restricted to this interval, little absorption-free baseline would be available. A wider scan can instead be used to estimate the baseline before integrating over a narrower interval. Including all 129 HITRAN transitions within ±110 GHz of the line center, instead of the 30 transitions of the ±25 GHz channel, leaves the rounded ±25 GHz values unchanged and alters the other entries by at most 0.2 percentage points, so the additional weak transitions do not change the reported ranking. Both line lists are selected using reference state pressure-shifted centers and then held fixed.

### 6.4. Baseline Error Sensitivity of the DAS Retrieval

The ideal DAS calculation presumes an exact baseline. To test how strongly this assumption affects the result, deterministic perturbations ΔA(ν) were added to the measured absorbance while the reference spectrum was left unchanged. Five shapes were considered, all with the same scale ε = 1 × 10^−3^ absorbance units, about 1.3% of the reference peak: a constant offset, Δ*A* = *ε*; a linear tilt, Δ*A* = *ε*(*ν* − *ν_c_*)/*Ω*; a quadratic curvature, $\Delta A\;=\;\varepsilon {\left[(\nu \;-\;\nu_{c})/\Omega \right]}^{2}$, which vanishes at the line center and reaches ε at the window edges ±Ω; and even and odd etalon-like ripples, Δ*A* = *ε* cos[2*π*(*ν* − *ν_c_*)/*Λ*] and Δ*A* = *ε* sin[2*π*(*ν* − *ν_c_*)/*Λ*], with free spectral range Λ. Because the retrieval in Equation (24) is a ratio to a fixed reference area, an additive perturbation shifts the retrieved value at every operating point by the same amount,

(25) $$\Delta \left(X_{{\mathrm{CH}}_{4}}\cdot L\right)=\frac{{\left(X_{{\mathrm{CH}}_{4}}\cdot L\right)}_{\mathrm{ref}}\int_{\nu_{c}-\Omega }^{\nu_{c}+\Omega }\Delta A(\nu )\mathrm{d\nu}}{A_{\mathrm{int},\mathrm{ref}}}$$

This shift is independent of temperature and pressure under the prescribed additive residual model. Table 2 lists the resulting shifts for half-widths Ω = 25 GHz and 10 GHz. The constant offset produces the largest change, 10.9 percentage points at ±25 GHz, and the quadratic curvature about one-third of that. The linear tilt and the odd ripple integrate to zero over the symmetric window, and the shift caused by the even ripple is bounded by 100 *εΛ*/(*π A*_int,ref_) percentage points, approximately 0.4 points for Λ = 5 GHz and 1.4 points for Λ = 20 GHz at Ω = 25 GHz. For Λ = 5 GHz, both windows in Table 2 contain an integer number of periods and the actual cosine contribution is zero; for Λ = 20 GHz at Ω = 25 GHz, it is +1.4 percentage points. These perturbations represent the residual left by an imperfect baseline reconstruction rather than the reconstruction itself; the low-order residual of a polynomial fit to absorption-free wings, for example, is captured by the constant and quadratic cases, whereas a particular reconstruction algorithm and its scan span or baseline regions are outside the present sensitivity analysis. In practical backscatter measurements, baseline reconstruction may therefore contribute more error than the finite integration window.

## 7. Overall Results and Discussion

Figure 15 brings the three retrievals together under the same forward model and reference calibration. Their relative performance depends on how temperature and pressure vary; none of the methods has the smallest uncompensated bias in every scenario.

FWMS gives the smallest endpoint error in both single-parameter sweeps. Its bias runs from +23.4% to −9.8% as temperature changes at constant pressure, and from −5.2% to −0.8% as pressure changes at constant temperature. The ranking changes for the closed volume, where DAS has the lowest endpoint bias, from +19.1% to −8.0%.

The RMSE values in Figure 15, calculated over every simulated point in each sweep, lead to the same general picture. Each scenario is evaluated on its own grid: 13 temperature points at 5 °C spacing for the constant-pressure and closed-volume sweeps, and nine pressure points at 0.05 atm spacing for the constant-temperature sweep, giving 35 operating points in total. For constant pressure, the RMSE is 11.9% for FWMS, 15.9% for WMS, and 18.2% for DAS. The corresponding constant-temperature values are 2.2%, 4.5%, and 15.7%. In the closed volume, DAS performs best at 9.7%, followed by FWMS at 12.3% and WMS at 14.6%. Combining all 35 operating points gives overall RMSE values of 10.5%, 13.4%, and 14.9% for FWMS, WMS, and DAS, respectively.

These differences arise because the methods sample the same absorption line in fundamentally different ways. Scanned WMS follows the harmonic response across a wavelength ramp and can accommodate a modest line center shift. The FWMS implementation considered here uses one harmonic metric at a fixed carrier. Across its modulation interval, pressure broadening partly offsets the change in number density, but any laser detuning appears directly in the retrieval. Ideal DAS integrates the full feature and is therefore insensitive to Voigt width and pressure shift; its area still depends on the line intensity and number density, and in practice, it is especially vulnerable to baseline error.

Within the assumptions tested here, FWMS is attractive when update rate and weak pressure sensitivity are priorities, whereas scanned WMS provides information on line center motion. DAS offers a line area metric when a sufficiently wide scan and a stable baseline are available. These observations guide instrument design but do not establish performance after device-specific correction and noise are included. The comparison emphasizes the need to address temperature and pressure explicitly for each retrieval.

### 7.1. Sensitivity of the Ranking to the Modulation Index

The modulation index m = 2.2 maximizes the second harmonic amplitude of a Lorentzian line at the reference state, but the linewidth changes from approximately 2.91 to 4.39 GHz across the operating envelope (Section 3.1 and Section 3.2), so the effective index a/HWHM already varies between 2.0 and 3.0 within the sweeps. To test whether the nominal choice of m affects the ranking, the WMS and FWMS retrievals were recomputed for nominal indices between 1.6 and 3.0, i.e., FM depths a = 3.18–5.96 GHz, with every other parameter unchanged; DAS does not depend on m. Table 3 lists the resulting RMSE values. The pooled RMSE of FWMS stays within 10.3–11.3% and that of WMS within 13.2–13.6%, compared with 14.9% for DAS, so the pooled ranking FWMS < WMS < DAS holds over the entire range. The only scenario-level change occurs in the constant-temperature sweep, where scanned WMS becomes slightly better than FWMS for m ≤ 1.8 (1.5% versus 2.0% at m = 1.8), because the balance between the density increase and pressure broadening that flattens the harmonic response at constant temperature depends on how much of the line the modulation samples. Increasing m reduces the closed-volume RMSE of both harmonic methods over the tested range. In the constant-temperature sweep, the RMSE first reaches a minimum and then increases: the minima occur near m = 1.8 for WMS and m = 2.0 for FWMS. The conclusions of this section are therefore not specific to m = 2.2, although the absolute bias values are.

### 7.2. Relation to Field Compensation Practice and Instrument Effects

Common approaches to temperature and pressure compensation include auxiliary sensor correction, spectroscopic estimation, and data-driven models. In auxiliary sensor correction, co-located temperature and pressure readings are used with a lookup table or polynomial, as in mine safety, pipeline, and airborne methane sensors [11,12,15,34]. Figure 15 provides three one-dimensional sections of the model response at the specified methane fraction, path length, and laser parameters. These sections illustrate the correction required along the tested trajectories; they are not a complete two-dimensional T–P correction surface and need not transfer unchanged to other optical depths.

The contribution of auxiliary sensor error can be estimated locally. Let *b*(*T*,*P*) be the simulated percentage bias and *g*(*T*,*P*) = 1 + *b*(*T*,*P*)/100. For a multiplicative correction $C_{\mathrm{corr}}=C_{\mathrm{raw}}/g\left(\hat{T},\hat{P}\right)$, the first-order residual percentage error Δ_res_ is

(26) $$\Delta_{\mathrm{res}}≃-\frac{\frac{\partial b}{\partial T}\Delta T+\frac{\partial b}{\partial P}\Delta P}{1+\frac{b}{100}}$$

where $\Delta T\;=\;\hat{T}\;-\;T$ and $\Delta P\;=\;\hat{P}\;-\;P$. At the reference state, b = 0. The local slopes for WMS, FWMS, and DAS are approximately −0.65, −0.52, and −0.74% per kelvin and +13, −2.6, and +95% per atmosphere, respectively. Considering one sensor error at a time, bounds of ±1 K give approximately 0.5–0.7% temperature-related contributions; bounds of ±10 mbar give approximately 0.1%, 0.03%, and 0.9% pressure-related contributions for WMS, FWMS, and DAS. These are local first-order contributions, not total corrected measurement uncertainties; combining both errors requires their bounds or covariance as appropriate.

Spectroscopic compensation uses line shape information and sufficient independent observations or auxiliary constraints to estimate gas state parameters. Approaches include fitting scanned 2f/1f or absorbance profiles [9,28] and using several modulation depths at a fixed carrier [13]. A linewidth alone does not independently determine every gas state variable. The single-depth, single-metric FWMS configuration evaluated here does not independently identify pressure and concentration; the multi-depth approach in [13] provides additional observables. Data-driven compensation instead learns a correction from a calibration dataset [15]. The present simulations quantify the uncompensated model response that these approaches seek to correct, without evaluating their full experimental error budgets.

Practical instruments also encounter etalon fringes, laser frequency drift, residual amplitude modulation, detector noise and, in standoff geometries, speckle and target-dependent return [18,19,35,36,37]. Mitigation measures include wedged optics and fringe suppression algorithms, frequency locking [17], 1f normalization and background subtraction [5,38], and averaging. Their effectiveness depends on the instrument and measurement protocol. These effects can alter measured errors and rankings, whereas the present model isolates the calibration transfer contribution under stated assumptions. Section 6.4 offers a limited connection to baseline errors: a residual fringe represented as an additive absorbance perturbation enters the integrated area DAS retrieval through its signed area.

The reported rankings apply to the R(3) manifold near 1654 nm, the reference optical depth, the tested modulation depths and integration windows, and the adopted FM–IM amplitudes and phases. A different transition, optical depth, or laser tuning response may change the ordering; FM–IM parameters are device-specific quantities [29]. The pooled RMSE also reflects equal weighting of the 35 selected operating points, not a measured distribution of field conditions. Applying the absolute values to a particular instrument requires laser characterization and experimental assessment. Such validation would be a valuable extension beyond the present simulation study; the framework can be rerun with instrument-specific inputs.

## 8. Conclusions

This study compares scanned WMS, FWMS, and integrated area DAS for methane detection near 1654 nm using a common HITRAN2020 line-by-line spectrum, Beer–Lambert transmission model, laser parameters, double-pass geometry, and reference calibration. The comparison isolates the uncompensated model bias when a calibration established at 293 K and 1 atm is transferred to another thermodynamic state. It describes three specified retrieval metrics under controlled assumptions; experimental instrument performance also depends on noise, drift, gas state uncertainty, and correction procedures.

No retrieval was uniformly superior. FWMS produced the lowest bias for the constant-pressure and constant-temperature sweeps, whereas DAS performed best in the closed-volume case. At constant pressure, the RMSE values were 11.9%, 15.9%, and 18.2% for FWMS, WMS, and DAS; at constant temperature, they were 2.2%, 4.5%, and 15.7%. The closed-volume values were 12.3%, 14.6%, and 9.7%, respectively. When all simulated points were combined, FWMS had the lowest overall RMSE, 10.5%. The largest individual error, +36.8%, occurred for DAS at −20 °C and constant pressure.

The results demonstrate the need for explicit temperature and pressure compensation within the operating envelope considered. The pooled ranking is unchanged for modulation indices between 1.6 and 3.0 and DAS integration windows between ±10 and ±100 GHz, although absolute biases and rankings outside these tests may depend on the transition, optical depth, and laser-specific FM–IM parameters. Detector noise, speckle, target inhomogeneity, drift, and a specific experimental baseline reconstruction procedure are outside the model. The additive baseline residual analysis and local auxiliary sensor error estimates describe selected contributions rather than a complete instrument uncertainty budget. Experimental validation with a characterized laser would extend the applicability of this conditional numerical comparison.

## Figures and Tables

**Figure 1:**
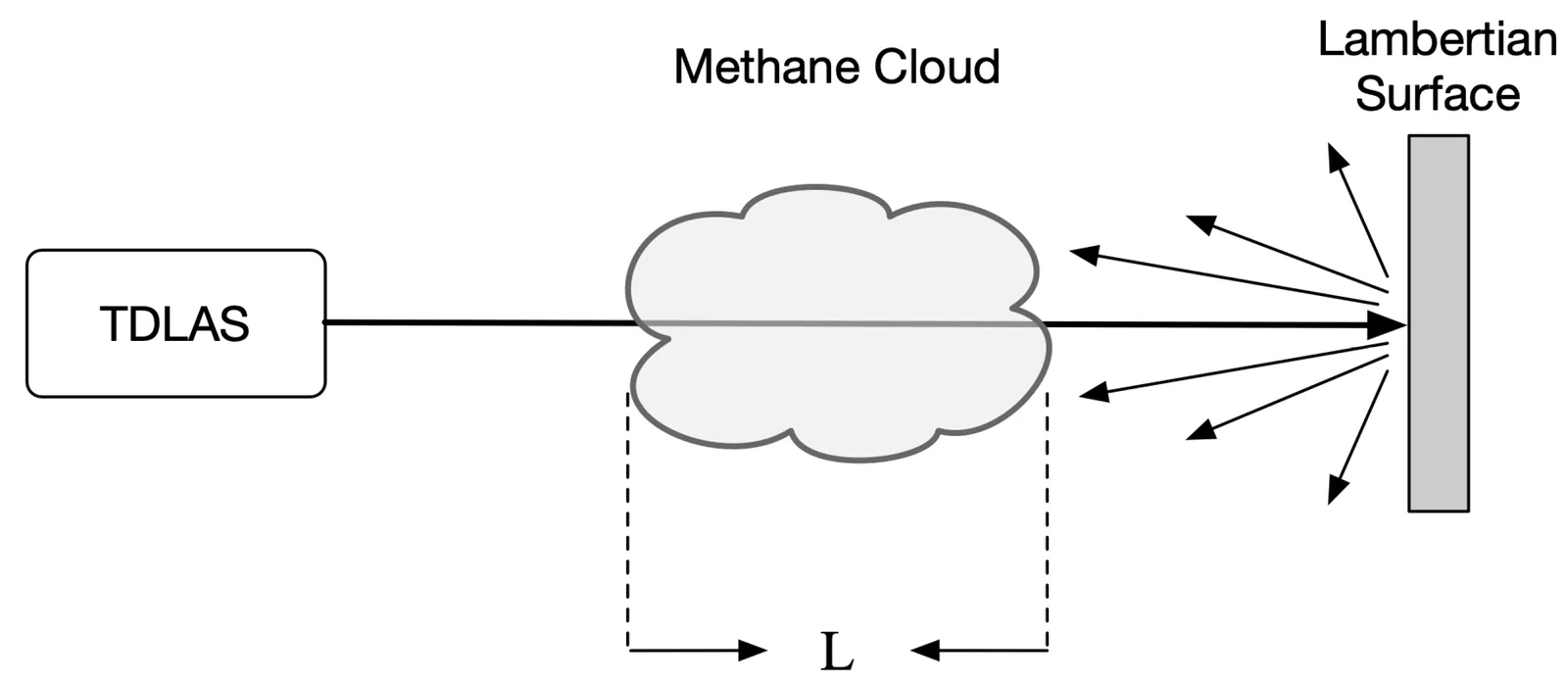
Schematic of the TDLAS open-path double-pass Lambertian backscatter geometry. The probe beam traverses the methane cloud of single-pass length L, reflects diffusely off the target surface, and returns through the cloud to a co-located photodetector, yielding a total optical path of 2 L.

**Figure 2:**
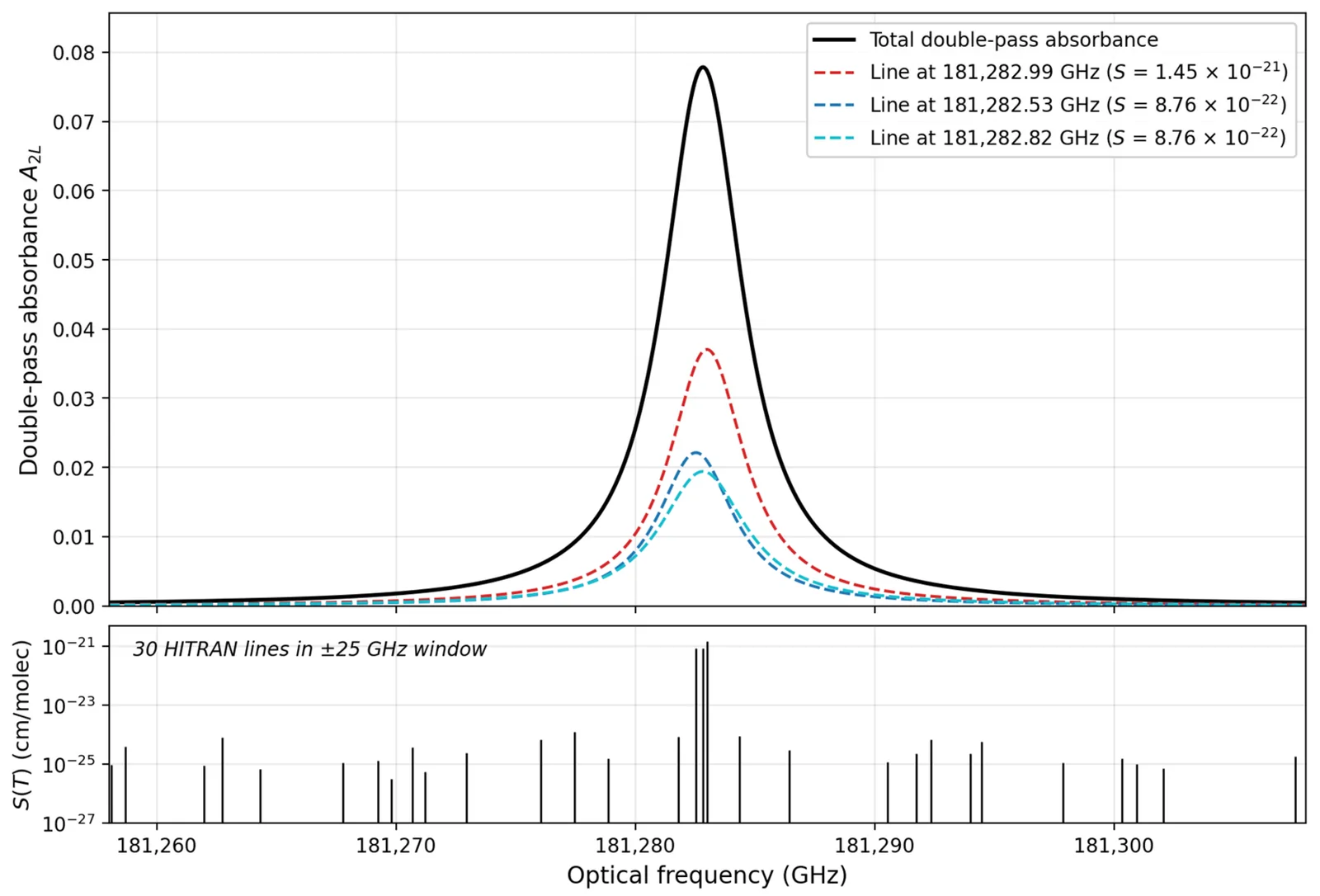
CH_4_ R(3) manifold around the calibration line center at 6046.944 cm^−1^ (181,282.82 GHz). Dashed curves show the three strongest individual Voigt contributions and the solid black curve is the total double-pass line-by-line absorbance at the reference state (T = 293 K, P = 1 atm, X_CH4_ = 1000 ppm, L = 1 m).

**Figure 3:**
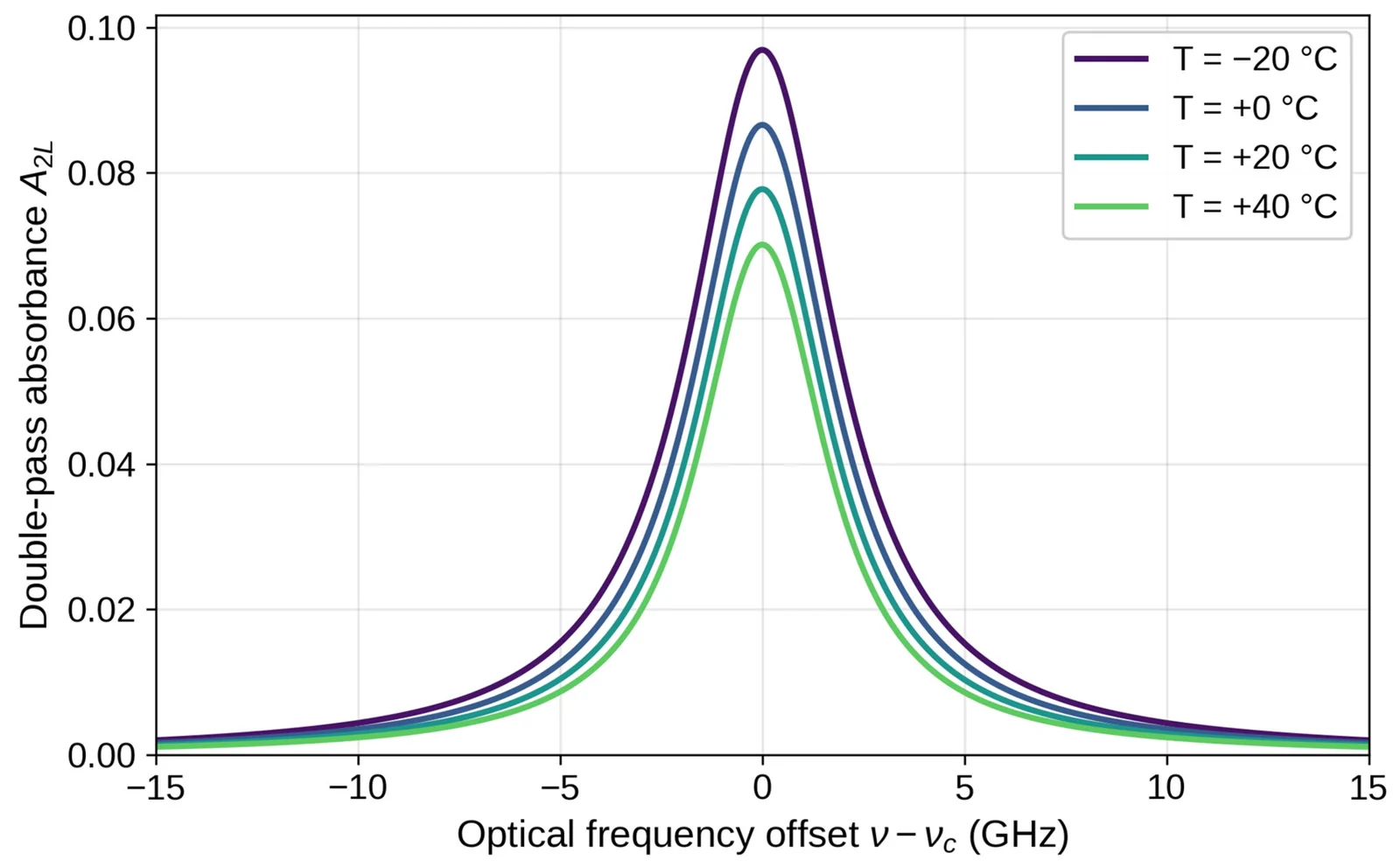
Absorption spectrum at four temperatures (T = −20, 0, 20, 40 °C) at P = 1 atm (constant pressure). Peak absorbance varies strongly with temperature, reaching its maximum at −20 °C. Frequency axis offset is from the calibration line center ν_c_ = 181,282.82 GHz (6046.944 cm^−1^) of the CH_4_ R(3) manifold.

**Figure 4:**
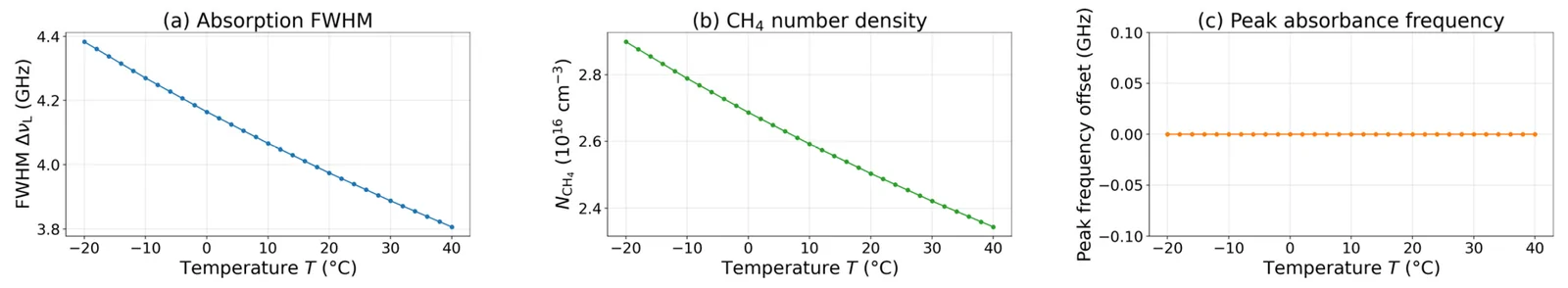
Thermodynamic trends of the CH_4_ R(3) line at constant pressure (P = 1 atm) over T = −20 to +40 °C. (**a**) Absorption FWHM, (**b**) CH_4_ number density, and (**c**) peak absorbance frequency versus temperature.

**Figure 5:**
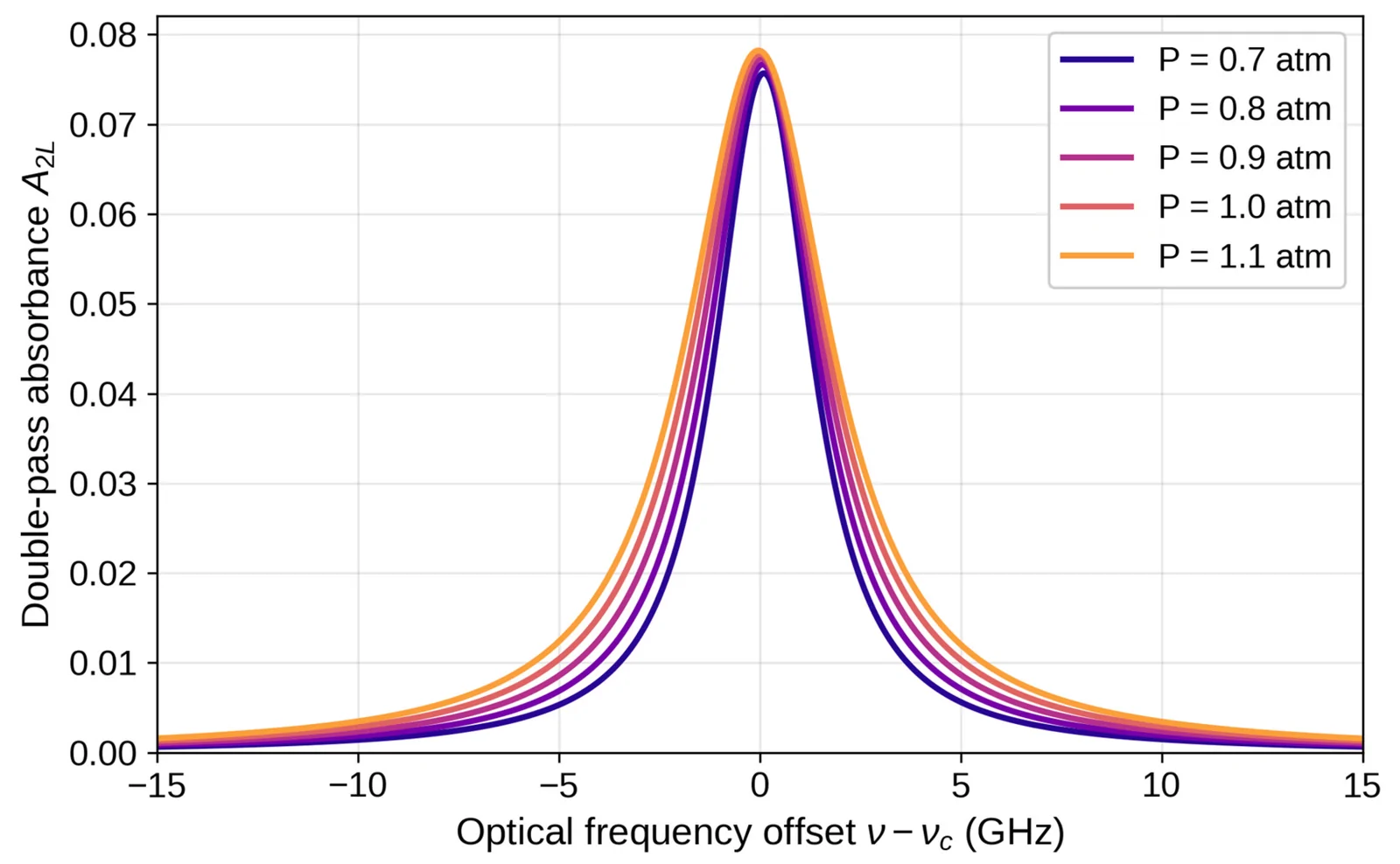
Absorption spectrum at five pressures (P = 0.7–1.1 atm) at T = 293 K. Peak absorbance is nearly constant while the wings fill in with rising pressure. Frequency axis offset is from the calibration line center ν_c_ = 181,282.82 GHz (6046.944 cm^−1^) of the CH_4_ R(3) manifold.

**Figure 6:**
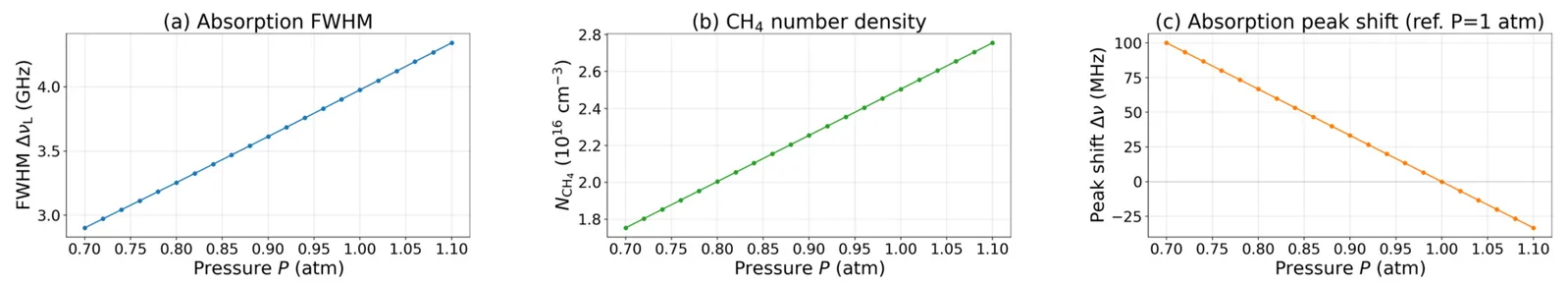
Thermodynamic trends of the CH_4_ R(3) line at fixed temperature (T = 293 K) over the pressure range 0.7–1.1 atm. (**a**) Absorption FWHM, (**b**) CH_4_ number density, and (**c**) absorption peak shift relative to P = 1 atm versus pressure.

**Figure 7:**
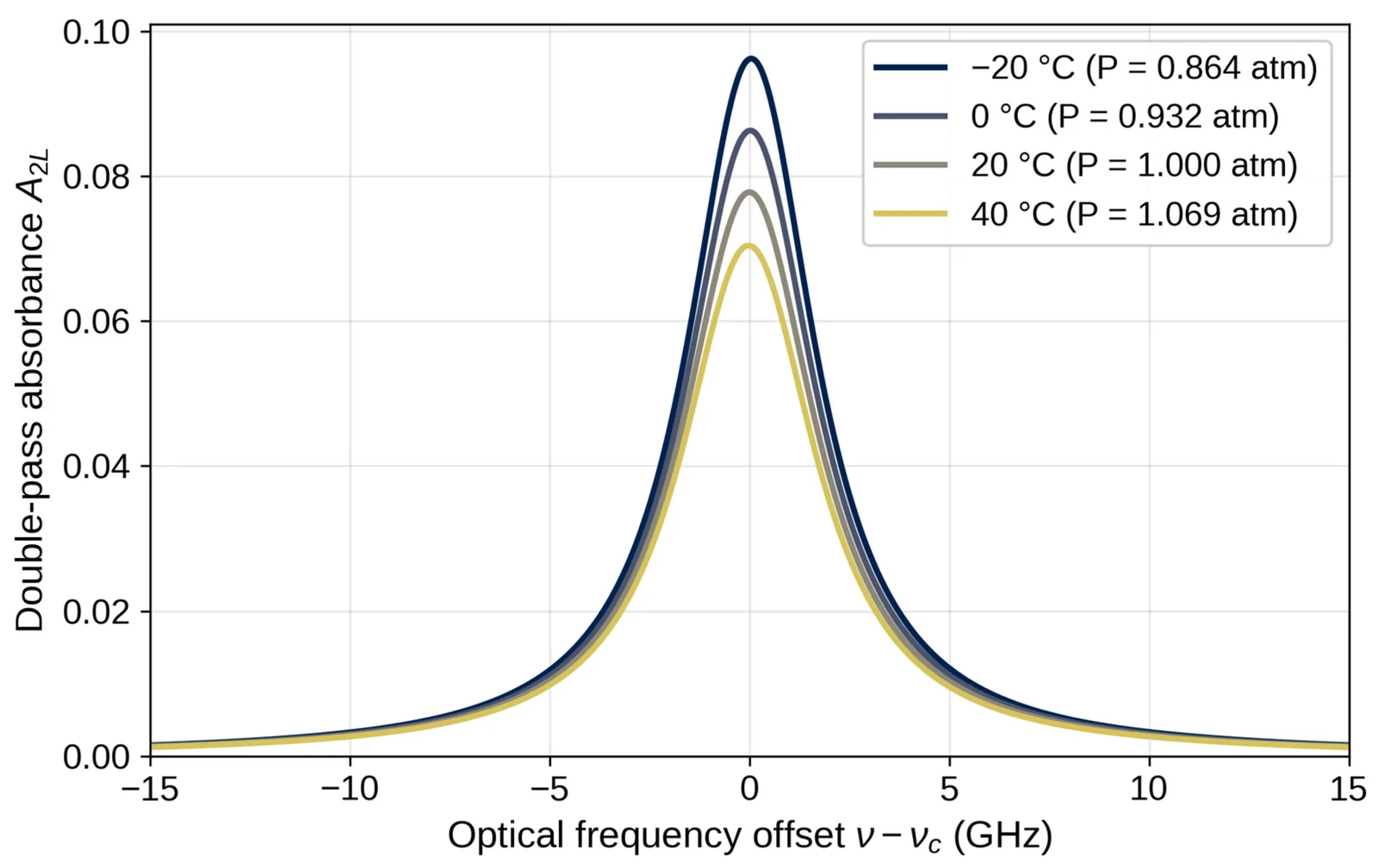
Absorption spectrum in a closed volume at four coupled (T, P) operating points spanning T = −20 to +40 °C with *P*(*T*) = *P*_ref_ · *T*/*T*_ref_. The frequency axis is the offset from the calibration line center of the CH_4_ R(3) manifold, ν_c_ = 181,282.82 GHz (6046.944 cm^−1^).

**Figure 8:**
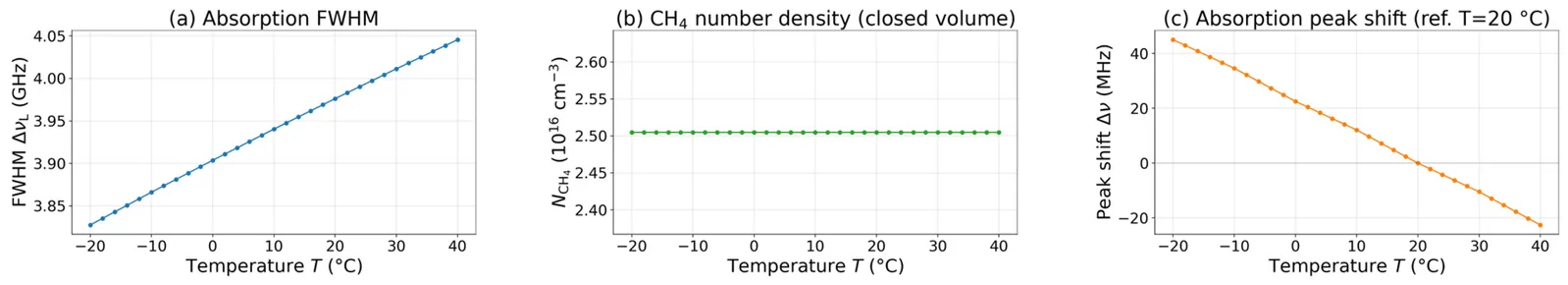
Thermodynamic trends in the closed-volume scenario, *P*(*T*) = *P*_ref_ · *T*/*T*_ref_, over T = −20 to +40 °C. (**a**) Absorption FWHM, (**b**) CH_4_ number density (exactly constant by construction), and (**c**) absorption peak shift relative to T = 293 K versus temperature.

**Figure 9:**
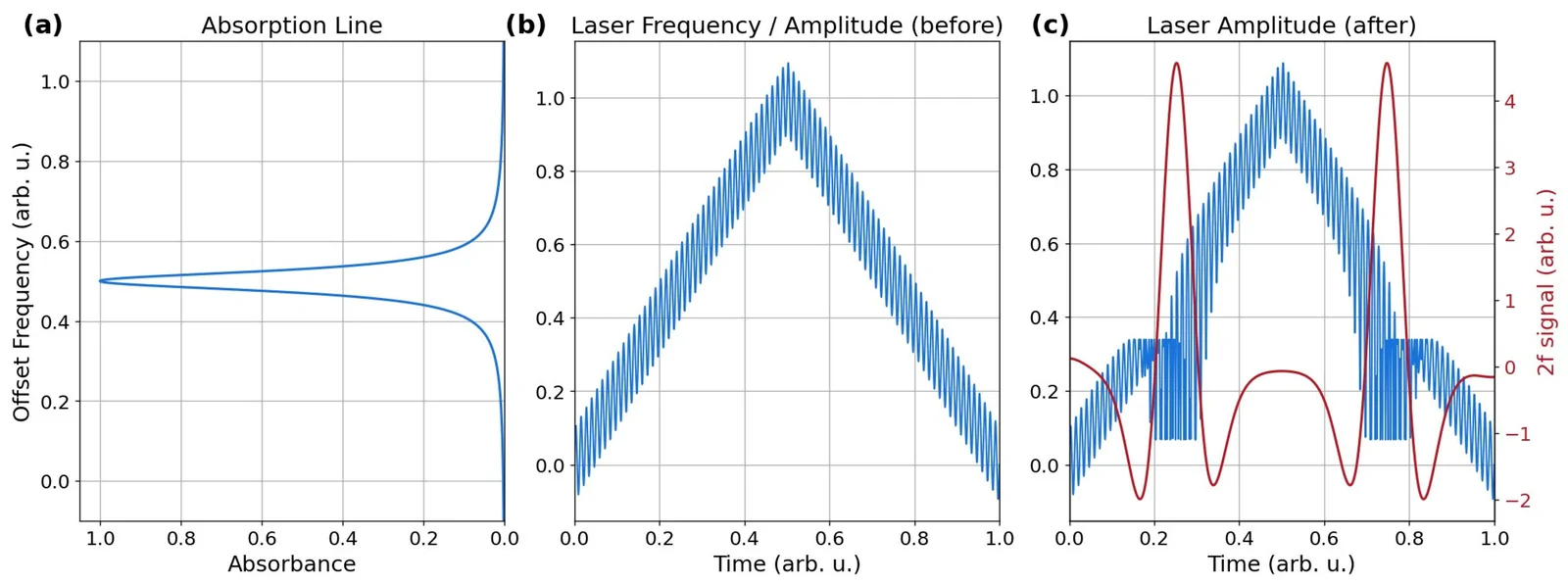
Working principle of scanned WMS on a single Voigt line. (**a**) Absorption profile A(ν); (**b**) instantaneous laser frequency before the absorbing gas; (**c**) transmitted laser amplitude after the absorbing gas (blue, left axis) and the corresponding signed in-phase 2f lock-in component X_2f_(ν_0_) (red, right axis).

**Figure 10:**
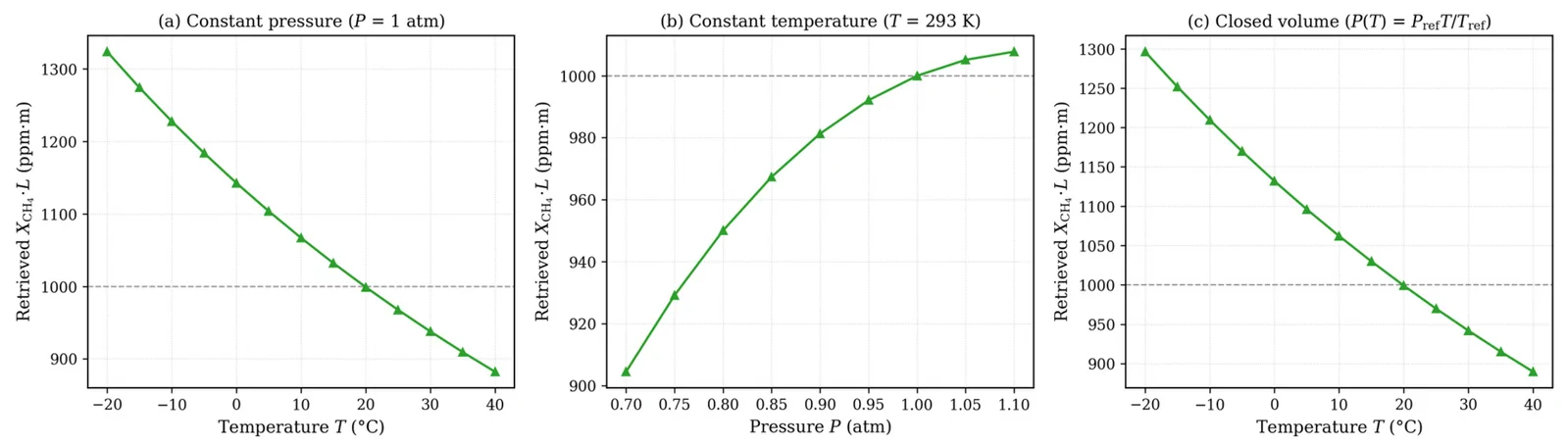
Scanned WMS-2f/1f retrieval across the three thermodynamic scenarios: (**a**) constant-pressure case (P = 1 atm) with temperature scan from –20 to +40 °C; (**b**) constant-temperature case (T = 293 K) with pressure scan from 0.7 to 1.1 atm; (**c**) closed-volume case with coupled pressure–temperature variation *P*(*T*) = *P*_ref_ · *T*/*T*_ref_. In all panels, the y-axis gives the retrieved path-integrated concentration in ppm·m (dashed gray reference at 1000 ppm·m).

**Figure 11:**
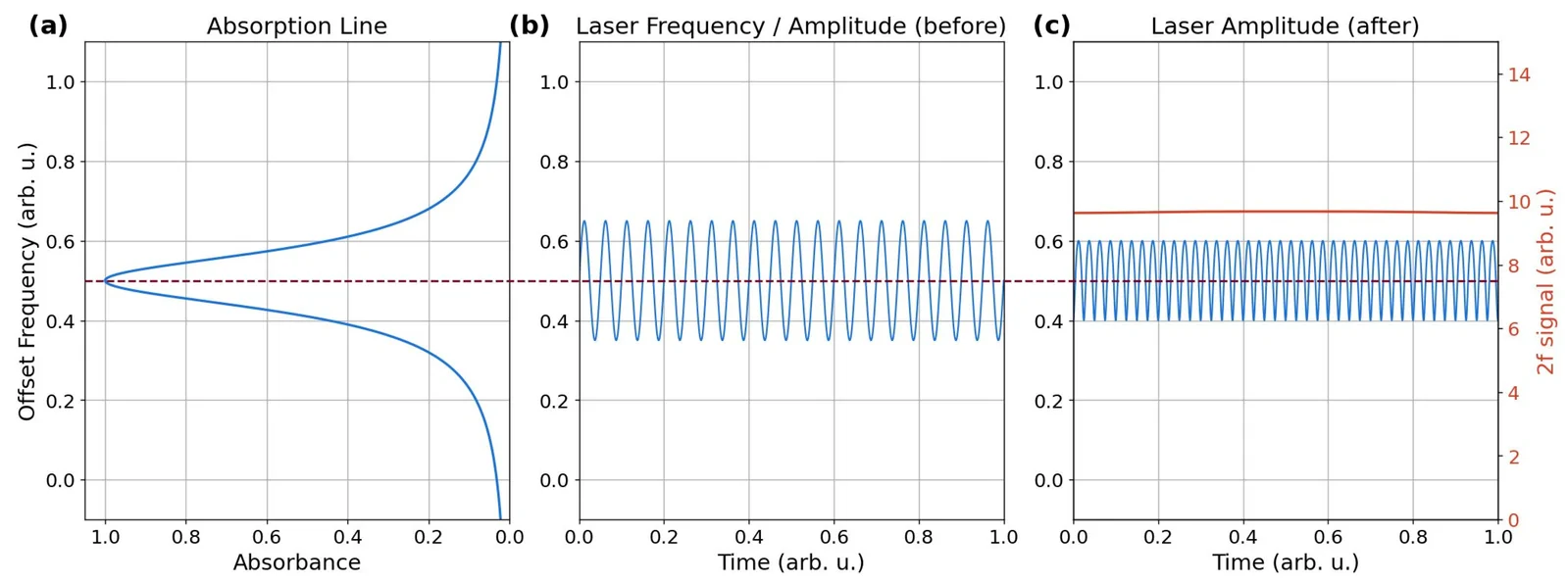
Working principle of fixed-wavelength modulation spectroscopy (FWMS) on a single Voigt line. (**a**) Absorption profile A(ν) with the laser carrier fixed at *ν* = *ν_c_* (dashed red); (**b**) instantaneous laser frequency before the absorbing gas; (**c**) transmitted laser amplitude after the absorbing gas (blue, left axis) and the corresponding 2f lock-in signal R_2f_(t) (red, right axis).

**Figure 12:**
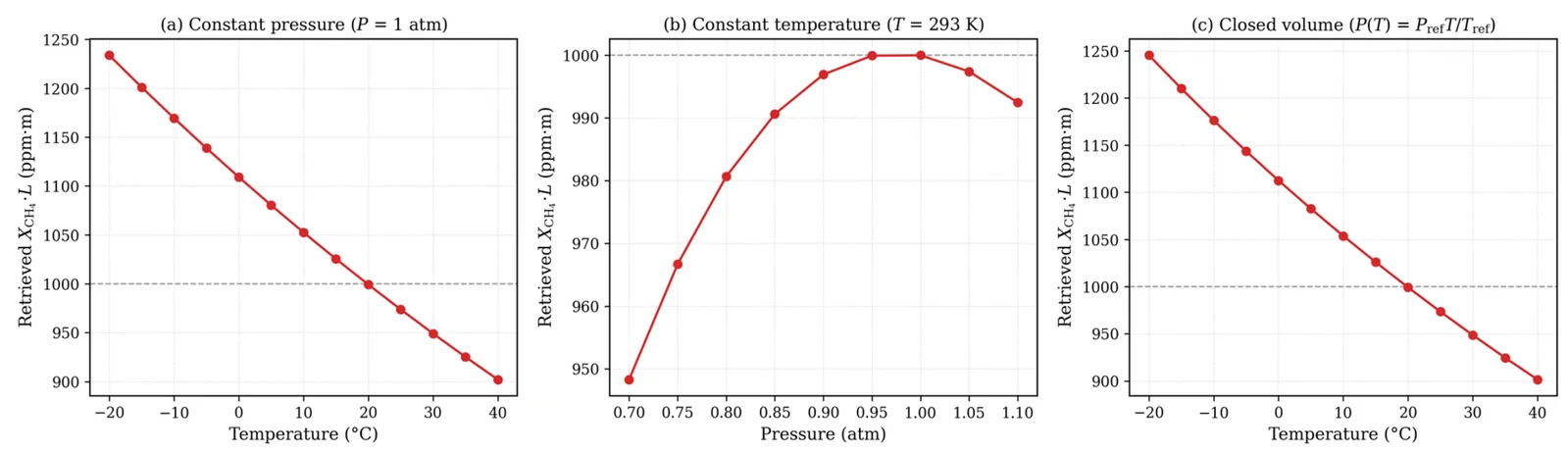
FWMS-2f/1f retrieval across the three thermodynamic scenarios: (**a**) constant-pressure case (P = 1 atm) with temperature scan from –20 to +40 °C; (**b**) constant-temperature case (T = 293 K) with pressure scan from 0.7 to 1.1 atm; (**c**) closed-volume case with coupled pressure–temperature variation *P*(*T*) = *P*_ref_ · *T*/*T*_ref_. In all panels, the y-axis gives the retrieved path-integrated concentration in ppm·m (dashed gray reference at 1000 ppm·m).

**Figure 13:**
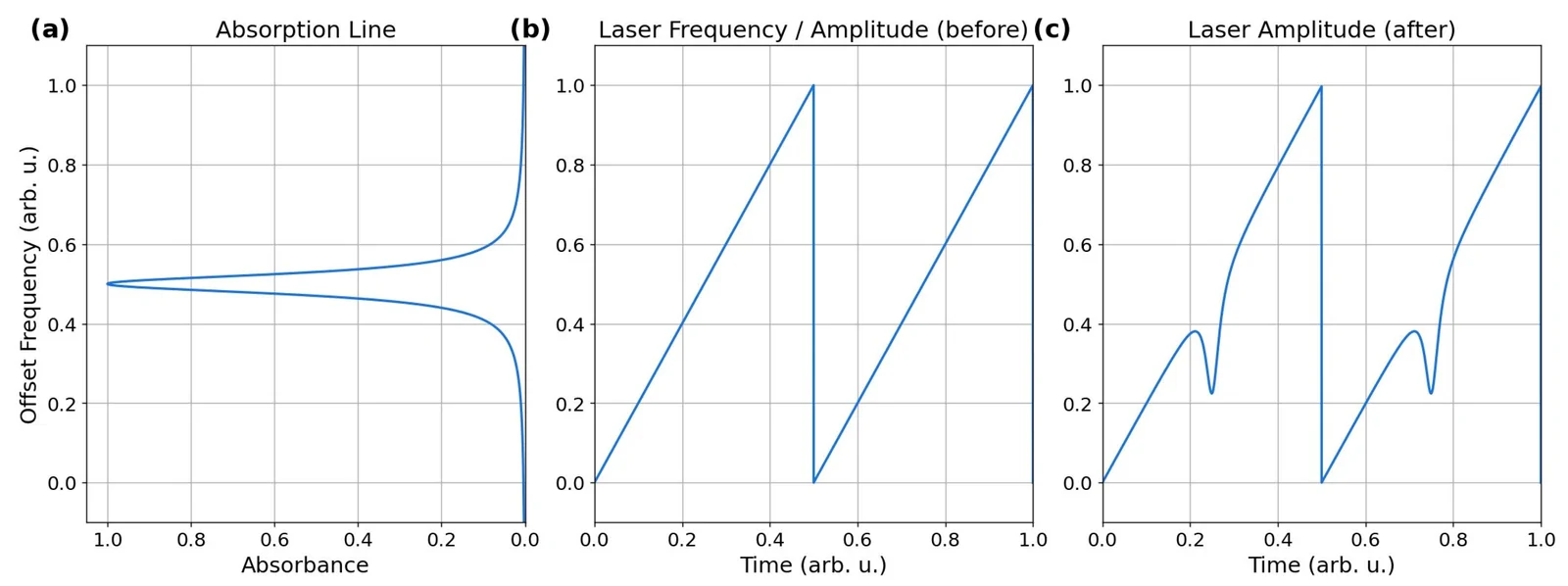
Working principle of direct absorption spectroscopy (DAS) with integrated area retrieval, illustrated on a single Voigt absorption line. (**a**) Absorption line profile A(ν). (**b**) Instantaneous laser frequency before the absorbing gas. (**c**) Transmitted laser amplitude after the absorbing gas.

**Figure 14:**
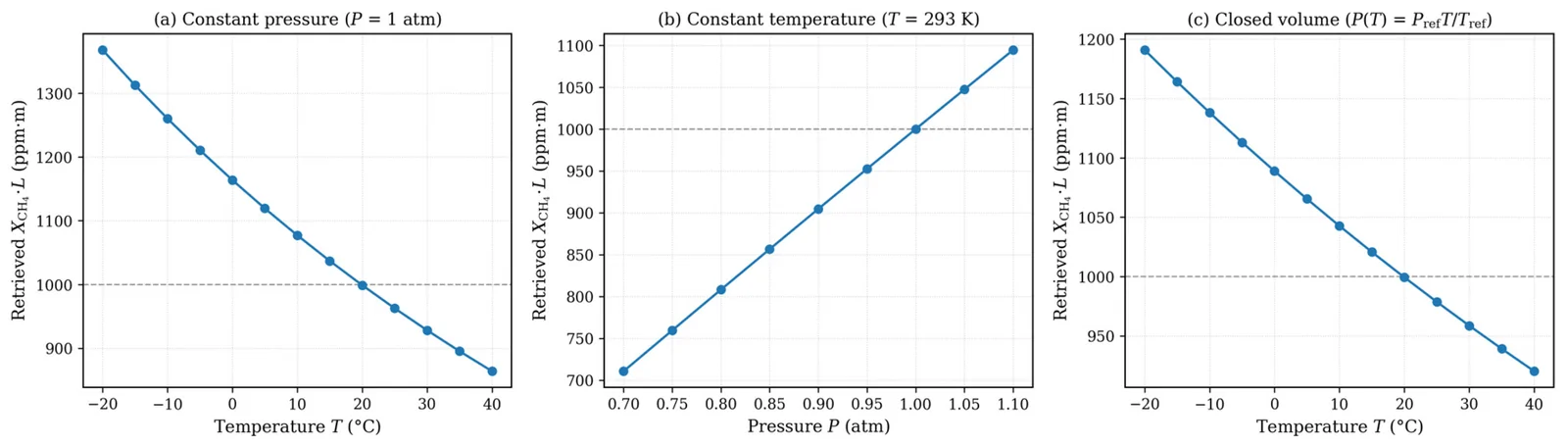
DAS integrated area retrieval across the three thermodynamic scenarios: (**a**) constant-pressure case (P = 1 atm) with temperature scan from –20 to +40 °C; (**b**) constant-temperature case (T = 293 K) with pressure scan from 0.7 to 1.1 atm; (**c**) closed-volume case with coupled pressure–temperature variation *P*(*T*) = *P*_ref_ · *T*/*T*_ref_. In all panels, the y-axis gives the retrieved path-integrated concentration in ppm·m (dashed gray reference at 1000 ppm·m).

**Figure 15:**
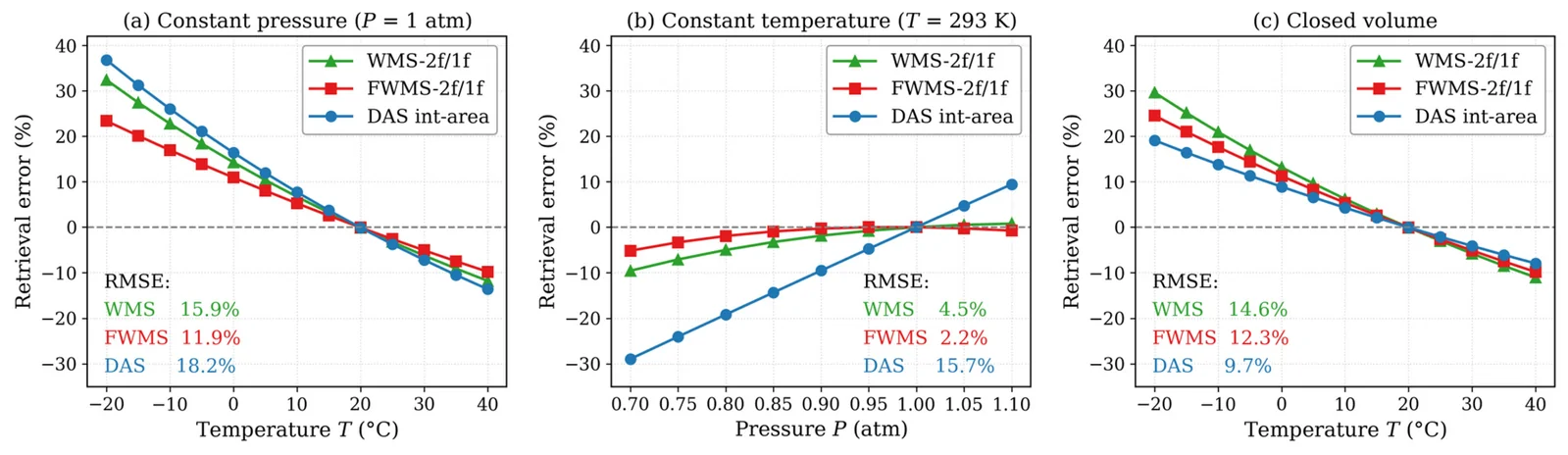
Head-to-head comparison of retrieval errors for scanned WMS-2f/1f (green triangles), FWMS-2f/1f (red squares), and DAS integrated area (blue circles) across the three thermodynamic scenarios: (**a**) constant-pressure temperature scan, (**b**) constant-temperature pressure scan, (**c**) closed volume. The RMSE of each retrieval over the scan grid of the corresponding scenario is annotated in each panel. All three panels use the same vertical scale.

**Table 1:** DAS integrated area retrieval error as a function of the integration half-width Ω. The second column gives the reference state area A_int,ref_ relative to its ±100 GHz value. RMSE values are listed for the constant-pressure (CP), constant-temperature (CT), and closed-volume (CV) sweeps and for the pooled 35 operating points.

Ω (GHz)	A_int,ref_ (% of ±100 GHz)	RMSE CP (%)	RMSE CT (%)	RMSE CV (%)	RMSE Pooled (%)
5	77.3	16.6	12.7	10.3	13.5
10	88.9	17.6	14.6	9.9	14.4
15	92.9	17.9	15.2	9.8	14.6
20	95.0	18.1	15.5	9.7	14.8
25	96.3	18.2	15.7	9.7	14.9
35	97.7	18.3	15.9	9.7	15.0
50	98.8	18.4	16.0	9.6	15.0
100	100	18.5	16.2	9.6	15.1

**Table 2:** Change in the DAS calibration transfer bias, in percentage points, produced by additive baseline perturbations of amplitude ε = 1 × 10^−3^ (1.3% of the reference peak absorbance) for two integration half-widths Ω. Because the retrieval is a ratio to a fixed reference area, the shift is identical at all 35 operating points. For the even ripple, the entry is the upper bound 100 *εΛ*/(*π A*_int,ref_); the exact value is zero whenever the window contains an integer number of ripple periods.

Perturbation	Definition of ΔA(ν)	Ω = 25 GHz	Ω = 10 GHz
Constant offset	*ε*	+10.9	+4.7
Linear tilt	*ε*(*ν* − *ν_c_*)/*Ω*	0.0	0.0
Quadratic curvature	$\varepsilon {\left[(\nu -\nu_{c})/\Omega \right]}^{2}$	+3.6	+1.6
Even ripple (Λ = 5 GHz)	*ε* cos[2*π*(*ν* − *ν_c_*)/*Λ*]	≤0.4	≤0.4
Odd ripple (Λ = 5 GHz)	*ε* sin[2*π*(*ν* − *ν_c_*)/*Λ*]	0.0	0.0

**Table 3:** RMSE (%) of the WMS and FWMS retrievals for nominal modulation indices m (FM depth a) between 1.6 and 3.0, listed for the constant-pressure (CP), constant-temperature (CT), and closed-volume (CV) sweeps and for the pooled 35 operating points. The m = 2.2 row reproduces Figure 15. DAS is independent of m (pooled RMSE 14.9%).

m	a (GHz)	CP WMS	CP FWMS	CT WMS	CT FWMS	CV WMS	CV FWMS	Pooled WMS	Pooled FWMS
1.6	3.18	14.1	9.8	1.7	4.3	16.3	13.5	13.2	10.4
1.8	3.58	14.9	10.6	1.5	2.0	15.7	13.1	13.2	10.3
2.0	3.98	15.5	11.3	3.1	1.1	15.2	12.7	13.3	10.4
2.2	4.37	15.9	11.9	4.5	2.2	14.6	12.3	13.4	10.5
2.4	4.77	16.2	12.4	5.7	3.5	14.2	12.1	13.4	10.7
2.6	5.17	16.5	12.8	6.6	4.6	13.8	11.8	13.5	10.9
2.8	5.57	16.6	13.2	7.4	5.5	13.4	11.6	13.5	11.1
3.0	5.96	16.7	13.5	8.1	6.3	13.1	11.4	13.6	11.3

## Data Availability

Data underlying the results presented in this paper (the HITRAN2020 CH_4_ line list extract, model parameters, and simulated absorbance and retrieval curves for the three thermodynamic scenarios) can be obtained from the corresponding author upon reasonable request.
